# Effect of Temperature, Salinity, and pH on Nanocellulose-Improved Polymer Gel for Oilfield Water Control

**DOI:** 10.3390/gels11030151

**Published:** 2025-02-20

**Authors:** Previnah Loganathan, Harjeet Kaur Sukhbir Singh, Zulhelmi Amir

**Affiliations:** 1 Department of Chemical Engineering, Faculty of Engineering, Universiti Malaya, Kuala Lumpur 50603, Malaysia; previnah@gmail.com (P.L.);; 2 Sustainable Process Engineering Center (SPEC), Department of Chemical Engineering, Faculty of Engineering, Universiti Malaya, Kuala Lumpur 50603, Malaysia

**Keywords:** water control, polymer gel, cellulose nanofibrils (CNF), polyacrylamide (PAM), gelation time, gel strength

## Abstract

Excessive water produced in oil reservoirs reduces oil recovery and increases the cost of water treatment. Conventional water control methods use synthetic polymer gels like PAM-PEI, which are sensitive to harsh reservoir conditions. This study investigates the use of cellulose nanofibers (CNF) to enhance polymer gels for oilfield water control under various temperatures, salinities, and pH conditions. Polymer gels were prepared by combining PAM and PEI with CNF concentrations of 1–4 wt% in deionized water. Salinity effects were studied by adding NaCl (1.5–2.5 g), while pH effects were assessed under acidic (pH 2–3), neutral, and alkaline (pH 13–14) conditions. The mixtures were stirred, homogenized, and subjected to thermal treatment in a water bath oven at temperatures ranging from 70 °C to 90 °C for gel formation. Gelation time was determined by the Sydansk gel code, and gel strength was assessed through storage modulus (G′) and loss modulus (G″) from oscillatory rheometry tests. Results show that lower temperatures increase gelation time, with higher CNF concentrations needed to elongate gelation at higher temperatures. At 30,000 ppm NaCl, gelation time decreases with increasing CNF, while at 50,000 ppm NaCl, it increases. Extreme pH conditions (pH 2–3 and pH 13–14) lead to longer gelation times with decreasing CNF concentration. While high salinity and extreme pH reduce gel strength, the addition of CNF enhances it, though this effect is minimal beyond 2–3 wt%. These findings suggest that CNF can improve the performance of polymer gels under challenging reservoir conditions.

## 1. Introduction

Excessive water production is a persistent challenge in the oil and gas industry, particularly in mature reservoirs where water–oil ratios (WORs) often exceed 4:1 and, in some cases, even reach values as high as 7:1 [1]. This excessive water reduces oil recovery efficiency, increases operational costs, and places additional strain on water treatment systems. Globally, managing produced water costs the oil and gas industry over USD 40 billion annually, with significant environmental impacts due to energy-intensive treatment and disposal processes [2]. The water’s high salinity, organic contaminants, and other impurities complicate its management, further emphasizing the importance of effective water control strategies.

Waterflooding, which is currently the most widely utilized secondary oil recovery technique, was initially employed to sustain pressure after primary depletion [3]. When water is injected into a reservoir, it travels fastest through areas with maximum permeability. Consequently, water in these high-permeability zones bypasses oil-rich areas, leading to increased water production and leaving significant volumes of un-swept oil in lower-permeability zones. This results in low oil sweep efficiency and higher operational costs for water treatment, reducing overall profitability for oil companies [4].

These conditions allow water to bypass oil-rich zones, leaving large volumes of recoverable hydrocarbons untapped. Such challenges have led to the widespread adoption of polymer gels, which selectively reduce permeability in water-saturated zones while maintaining oil flow pathways. These gels are particularly effective in heterogeneous reservoirs and are commonly used in conformance control applications [5]. Synthetic polymer gels, such as those based on polyacrylamide (PAM) cross-linked with polyethyleneimine (PEI), are among the most widely used systems for water shutoff applications. These gels form three-dimensional networks that block water flow through high-permeability channels, improving sweep efficiency and oil recovery [6]. However, under harsh reservoir conditions, including high salinity, extreme pH, and elevated temperatures, these gels face significant challenges. High salinity, for instance, compresses the electric double layer of polymer molecules, reducing their ability to form strong cross-linked networks [7]. Similarly, extreme pH conditions can destabilize the gel structure, while high temperatures often accelerate degradation, leading to reduced mechanical strength and effectiveness [8].

Recent advancements in enhancing the efficiency of gelling agents for high-salinity and complex reservoir conditions have focused on incorporating various additives that improve the gels’ stability and performance. One significant improvement involves the use of nano silica in polymer gels. Nano silica particles, due to their high surface area and small particle size, interact with the polymer matrix, creating a stronger and more stable gel network. This interaction increases the gel’s ability to retain bound water, which is crucial for maintaining gel integrity in harsh reservoir conditions. Nano silica enhances the thermal and salinity resistance of the gel by reinforcing the structure, thus preventing degradation under high-temperature and high-salinity conditions. This modification leads to improved mechanical properties, such as increased viscosity and shear strength, making the gels more effective for conformance control and water shutoff applications, particularly in deep and complex reservoirs where traditional gels may fail [9]. Additionally, the incorporation of cellulose nanofibrils (CNF) as a reinforcement material takes advantage of the high aspect ratio and surface area of CNF. These nanofibrils form a fibrous network within the polymer gel, improving its mechanical strength and preventing collapse under pressure or temperature fluctuations. CNF reinforce the gel structure, making it particularly effective in low-temperature, high-salinity environments, where gels without such reinforcement may not maintain their desired properties. CNF’s ability to form strong bonds with polymer chains enhances the gel’s stability and effectiveness, improving the overall performance in complex reservoir conditions [10].

Another approach to improving gel performance involves the addition of graphite to polymer gels. Graphite particles act as structural reinforcements within the polymer matrix, improving the thermal stability and mechanical strength of the gel. Graphite’s excellent thermal conductivity ensures that the gel can withstand high temperatures without losing its integrity, making it ideal for reservoirs with extreme heat conditions. Additionally, graphite improves the gel’s resistance to salinity by strengthening the polymer framework, allowing the gel to maintain its viscosity and stability in saline environments. This combination of enhanced thermal and salinity resistance extends the lifespan and effectiveness of the gel, particularly in reservoirs that experience fluctuating or extreme environmental conditions [9]. Oily sludge has also been explored as an additive in polymer gels, offering both a cost-effective and sustainable approach to improving gel performance. The organic components in oily sludge, such as hydrocarbons, contribute to the gel’s mechanical strength and help it maintain its stability in high-temperature and high-salinity conditions. When added to the polymer matrix, oily sludge enhances the gel’s viscosity and resistance to degradation, providing a viable solution for regions with abundant waste materials. This approach not only improves the gel’s performance but also provides an environmentally friendly alternative by utilizing waste products that would otherwise be discarded [9].

In addition to these materials, foam-enhanced polymer gels have emerged as a hybrid system that offers superior stability, injectability, and resistance to harsh reservoir conditions. The incorporation of foam into polymer gels helps to distribute the gel more evenly throughout the reservoir, ensuring better penetration and uniformity. Foam bubbles act as a stabilizing agent that prevents gel aggregation, ensuring that the gel remains fluid enough for efficient injection while still maintaining its gel-like consistency once in the reservoir. This hybrid system also enhances the gel’s structural integrity, allowing it to resist breaking down under high salinity or temperature conditions. Foam-enhanced gels are particularly useful for profile control and water shutoff treatments, where uniform distribution and the ability to remain stable under challenging conditions are crucial. By improving both the injectability and stability of the gel, foam-enhanced systems are particularly suited for complex reservoir environments where traditional gels may fail to provide the necessary performance [9].

In recent years, biopolymers such as nanocellulose have emerged as promising alternatives to enhance the performance of polymer gels under harsh conditions. Nanocellulose, derived from renewable plant-based sources, is a versatile material known for its high mechanical strength, biodegradability, and tunable surface properties [6]. It exists in several forms, including cellulose nanofibrils (CNF), cellulose nanocrystals (CNC), and bacterial nanocellulose (BNC), each with unique structural and functional attributes [11]. Nanocellulose has been shown to improve the viscoelasticity of polymer gels, increase their tolerance to high salinity and extreme pH, and enhance their overall stability and performance [12]. Its ability to form additional cross-linking points within the gel network makes it an excellent additive for strengthening polymer gels and mitigating the effects of harsh reservoir conditions [13].

Studies have demonstrated that nanocellulose-enhanced polymer gels exhibit superior mechanical properties compared to conventional systems. For example, nanocellulose can reinforce the gel matrix by forming hydrogen bonds with the polymer chains, creating a denser and more interconnected network [6]. The nanoscale dimensions and high aspect ratio of nanocellulose enable it to act as a bridging agent, further improving the structural integrity of the gel [11]. Despite these advantages, most research on nanocellulose has focused on its applications in packaging, construction, and composites, with limited studies addressing its role in polymer gels for oilfield water control. Furthermore, the impact of nanocellulose concentration on gelation kinetics and gel strength under varying reservoir conditions remains poorly understood.

This study aims to address these research gaps by systematically investigating the effects of cellulose nanofibrils (CNF) on the gelation time and gel strength of PAM-PEI polymer gels under different temperatures, salinity, and pH conditions. Specifically, it seeks to determine the optimal CNF concentration required to balance gel strength and gelation time, particularly in high-salinity and extreme pH environments. By examining the viscoelastic properties and structural integrity of these nanocellulose-enhanced gels, this research aims to provide new insights into their performance and potential as sustainable solutions for water control in oilfields. The findings of this study are expected to contribute to the development of next-generation polymer gels that are not only effective under harsh reservoir conditions but also align with the oil and gas industry’s growing focus on environmental sustainability and cost efficiency.

## 2. Results and Discussion

### 2.1. Gelation Time

The time taken for a solution to solidify into a rigid gel is known as the gelation time. In polymer gel systems, gelation time plays a crucial role in ensuring PAM and PEI cross-linking is delayed, providing oilfield operators enough time to inject the gelling solution and allow it to move into the designated region. It is critical to evaluate the gelation periods of PAM/PEI gels at various temperatures, salinity, and pH levels with varied CNF concentrations.

#### 2.1.1. Effect of Temperature

Table 1, Table 2 and Table 3 show the Sydansk code obtained from the gel at 70 °C, 80 °C, and 90 °C, respectively.

At 70 °C, it is evident that lower concentrations of CNF lead to a longer gelation time; 1 wt% CNF and 2 wt% CNF gel mixture becomes rigid at 72 h, compared to the 3 wt% CNF and 4 wt% CNF gel mixture, which becomes rigid at 48 h. The reason for this is that larger CNF content offers more cross-linking points, which encourages the development of a gel network. In general, gel formulation’s gelation period may be shortened by using more cellulose nanofibrils (CNF) than usual. This is due to the role of CNF as a reinforcing agent, which supports the production of gel and the overall network structure. The PAM-PEI polymer gel’s network’s density and interconnectivity may grow as CNF concentration grows, which could speed up gelation. It is also clear that lower temperatures, such as 70 °C, increase gelation time. Slower gel formation can result from lower temperatures that disrupt molecular mobility and decelerate cross-linking processes.

At 80 °C, it is shown that all the gel samples become rigid at 48 h. This is because faster gel formation can result from higher temperatures that encourage molecular mobility and accelerate cross-linking processes. It is also clear that the concentration of CNF still matters. At 24 h, 1 wt% CNF and 2 wt% CNF gel mixture are less rigid compared to the 3 wt% CNF and 4 wt% CNF gel mixture. Higher concentrations of CNF speed up gelation by offering more cross-linking points.

At 90 °C, however, it is shown that higher concentrations of CNF increase the gelation time at high temperatures. This is a key finding, as most reservoirs are at a high temperature. Usually, hydrogen bonds between the cellulose nanofibrils serve to stabilize CNF gels. Higher temperatures cause water molecules to become more mobile, which interferes with the creation of hydrogen bonds and slows the gelation process. This is due to the possibility that higher thermal energy may render the connections between CNF particles less robust, necessitating a longer formation period for the bonds that stabilize the gel structure; 1 wt% CNF and 2 wt% CNF gel mixtures become rigid at 48 h, compared to the 3 wt% CNF and 4 wt% CNF gel mixtures, which become rigid at 72 h. The gel precursor solution’s viscosity may considerably increase at high CNF concentrations. The CNF particles have a harder time moving and interacting with one another at higher viscosities, which slows the gelation process. Despite the high CNF concentration and high temperature, the gelation time was increased for this gel mixture.

**Table 1:** Sydansk code at 70 °C.

Time	1 wt% CNF	2 wt% CNF	3 wt% CNF	4 wt% CNF
24 h	E	E	H	H
48 h	H	H	I	I
72 h	I	I	I	I

**Table 2:** Sydansk code at 80 °C.

Time	1 wt% CNF	2 wt% CNF	3 wt% CNF	4 wt% CNF
24 h	E	F	H	H
48 h	I	I	I	I
72 h	I	I	I	I

**Table 3:** Sydansk code at 90 °C.

Time	1 wt% CNF	2 wt% CNF	3 wt% CNF	4 wt% CNF
24 h	I	I	E	H
48 h	I	I	H	H
72 h	I	I	I	I

#### 2.1.2. Effect of Salinity

The results of the gelation time are determined every 24 h for 3 days based on the Sydansk code. Table 4, Table 5 and Table 6 show the recorded transformation of the gel at salinity of 30,000 ppm NaCl, 40,000 ppm NaCl, and 50,000 ppm NaCl, respectively. At 30,000 ppm NaCl, as shown in Table 4, increasing the CNF concentration from 1 wt% to 4 wt% initially results in a stronger gel, code E to F, but higher concentrations do not significantly enhance rigidity further at the 24 h mark. As the observation period extends to 48 and 72 h, all concentrations gradually reach a similarly high rigidity of code H, appearing to have the same gelation time. This is because larger CNF content promotes the growth of a gel network by offering more cross-linking points. The concentration of CNF may increase along with the density and interconnectivity of the PAM-PEI polymer gel network, thereby speeding the gelation process.

According to Table 5, at 40,000 ppm NaCl, the gelation varies differently with CNF concentration, showing a more immediate influence of salinity on gelation time compared to lower salinity conditions. For example, at 24 h, lower concentrations of CNF provide stronger gels of code D and C, and by 72 h, the rigidity patterns appear mixed; 1 wt% and 2 wt% CNF presence in the gel system show a higher degree of rigidity compared to 3 wt% and 4 wt% CNF. It can also be said that the gelation time is longer at higher concentrations of CNF (3 wt% and 4 wt% CNF) than at lower concentrations of CNF.

At 50,000 ppm NaCl, the rate of gel rigidity formation is obviously slower than at 30,000 ppm and 40,000 ppm NaCl; except for the gel with 1 wt% CNF, which formed a highly deformable non-flowing gel of code F, the other gels only formed as moderately flowing gel by 72 h. The gel with 2 wt%, 3 wt%, and 4 wt% CNF required a longer gelation time than the gel with 1 wt% CNF, as tabulated in Table 6.

Across all salinities and concentrations of CNF, the gel generally increases in rigidity as the gelation time extends from 24 h to 72 h. This trend shows that longer gelation time allows more extensive network formation within the gels, leading to stronger gel structures. The PAM/PEI gel system has a much longer gelation time as the salinity increases from 30,000 ppm to 50,000 ppm NaCl. The rate of the gel samples turning rigid is faster in the salinity condition of 30,000 ppm NaCl compared to 40,000 ppm, followed by 50,000 ppm NaCl.

When CNF are present, the performance of PAM/PEI polymer gel is significantly affected by increasing the salinity of the polymer solution. The increased ionic strength due to higher salinity can shield the electrostatic interactions between charged polymer chains; in this case, PAM is anionic, and PEI is cationic. Higher salinity can reduce the electrostatic attraction between oppositely charged polymers, potentially increasing the gelation time [7]. The size of PAM changes to a smaller hydrodynamic radius with higher positive salt ions. The conformation, entanglements, and orientation of the polymer molecules vary as a result of interactions between the polymer and the salt ions. The strong counter ions compress the diffuse layer as the charge number of Na+ increases, resulting in a decrease in the diffusion layer’s thickness. PAM chains are fairly responsive to the ionic strength of the higher-salinity water due to their flexibility [4]. The polymer chains then assumed a coiled configuration. The molecules of the compacted polymer cannot be fully extended when they are in a coiled form.

Therefore, the coiled state polymer offers less possibility to form bonds, even when the concentration of CNF increases and encourages the polymer’s degree of hydrolysis. It makes CNF’s ability to screen the polymer chain less effective, making it unable to extend the gelation time. Hence, the gelation time increases as the concentration of CNF increases when the salinity increases as well.

The mixture’s total viscosity increases in proportion to the CNF concentration. High viscosity may cause the polymer chains to become less mobile and difficult to locate and create cross-links, which may account for the longer gelation durations [14]. The effect of increased viscosity may be apparent at greater salinities because of the additional ionic interactions that influence the system’s fluid dynamics.

CNF are highly capable of retaining water. Higher CNF concentration slows down the gelation process because more water is retained inside the CNF structure, limiting the quantity accessible to promote polymer chain movement and interaction. This impact becomes more severe in high salinity conditions because of changes in the water structure, which could result in a more heterogeneous gel network [14]. Due to the non-uniform cross-linking densities caused by this heterogeneity, the development of a cohesive gel network becomes more structurally complex, lengthening the gelation period.

**Table 4:** Sydansk code at 30,000 ppm NaCl.

Time	1 wt% CNF	2 wt% CNF	3 wt% CNF	4 wt% CNF
24 h	E	F	F	E
48 h	G	H	H	G
72 h	H	H	H	H

**Table 5:** Sydansk code at 40,000 ppm NaCl.

Time	1 wt% CNF	2 wt% CNF	3 wt% CNF	4 wt% CNF
24 h	D	C	C	C
48 h	F	E	C	D
72 h	F	F	D	E

**Table 6:** Sydansk code at 50,000 ppm NaCl.

Time	1 wt% CNF	2 wt% CNF	3 wt% CNF	4 wt% CNF
24 h	C	B	B	B
48 h	E	D	D	C
72 h	F	D	D	C

#### 2.1.3. Effect of pH

Table 7, Table 8 and Table 9 tabulate the results of the gel transformation at pH 2–3, pH 13–14, and neutral conditions, respectively. At extreme acidity of pH 2 to 3, tabulated in Table 7, the gelation behavior of the PAM-PEI polymer gel is notably influenced by the concentration of CNF. Lower concentrations (1% and 2%) result in less pronounced gelation, forming flowing or moderately flowing gels (‘C’ and ‘D’, respectively) at 24 h. As time progresses, lower concentrations (1% and 2%) develop into more substantial gels (‘D’ and ‘F’, respectively), while higher concentrations (3% and 4%) result in slightly deformable to highly deformable non-flowing gels (‘G′ and ‘H’, respectively) by 72 h. Over time, gelation becomes more consistent across all concentrations, with highly or slightly deformable non-flowing gels dominating.

Table 8 tabulates the results at extreme alkalinity of pH 13 to 14, where gelation appears to be consistent across all CNF concentrations. Within 24 h, no detectable gel to highly flowing gel formed regardless of CNF concentration. This stability persists at codes A and B over the observation period, with very little to no variation observed from 24 to 72 h.

At neutral conditions, as shown in Table 9, the gelation process shows a clear dependence on CNF concentration. Lower concentrations of CNF at 1 wt% and 2 wt% lead to the formation of rigid gels at 24 h itself, while higher concentrations of CNF at 3% and 4% result in barely flowing gel and slightly deformable non-flowing gel at the 24 h mark. Over time, gelation continues, with higher concentrations resulting in increasingly rigid gels with code H and I at 48 and 72 h, while lower concentrations remain in the rigid gel at code I. Higher CNF concentrations have been demonstrated to lengthen the gelation period.

Overall, the trend of the results shows that gelation time is generally faster at neutral conditions compared to extremely acidic or alkaline conditions. However, the addition of CNF at neutral conditions increases the gelation time, whereas at pH 2–3 and pH 13–14 conditions, increasing the concentration of CNF decreases the gelation time.

Polyacrylamide (PAM) is stable across a wide pH range but may hydrolyze at high pH (above 10), forming anionic groups that impact interactions with PEI and CNF. Polyethyleneimine (PEI), a polyelectrolyte with a high density of amine groups, exhibits different behaviors depending on pH. At pH 2–3, PEI is fully protonated (positively charged), while at neutral pH, it is partially protonated, and at pH 13–14, many amine groups are deprotonated, reducing charge density. CNF carries negatively charged surface groups, such as carboxylates, which are more dissociated at higher pH levels.

At neutral conditions, high protonation of PEI enhances its interaction with both PAM and the less negatively charged CNF, promoting effective cross-linking and potentially faster gelation. Here, increasing CNF concentration has a different effect. The charged PEI interacts less strongly with PAM, and the CNF also does not form as many physical cross-links as in acidic or alkaline conditions. The presence of higher CNF concentrations increases the viscosity of the solution, which can impede the mobility of polymer chains. This slower movement hinders the formation of an interconnected gel network, leading to an increase in gelation time. Therefore, in neutral conditions, higher CNF concentrations tend to slow down the gelation process by creating a more viscous medium that retards the dynamic interactions necessary for rapid gel formation.

At pH 2–3, moderate protonation of PEI results in intermediate interaction strength, leading to intermediate gelation times. This enhances its interaction with both PAM and CNF, which are less negatively charged due to the reduced dissociation of carboxylate groups on CNF. The increased concentration of CNF provides more cross-linking points, promoting a more interconnected network. This enhanced network formation accelerates the gelation process, resulting in faster gelation times. Higher CNF concentrations in acidic conditions thus effectively facilitate the gelation process by reinforcing the network structure and reducing the time needed to form a gel.

At pH 13–14, minimal protonation of PEI and high negative charge on CNF result in weaker interactions and increased repulsive forces, potentially slowing gelation compared to acidic and neutral conditions. In alkaline conditions, the amine groups in PEI are mostly deprotonated, significantly reducing the positive charge density. Meanwhile, the carboxylate groups on CNF are fully dissociated, resulting in increased negative charges. Although the reduced positive charge on PEI weakens its interaction with PAM, the highly charged CNF still promote physical cross-linking. Increased CNF concentration under these conditions aids in forming a more gel network despite the weaker PAM-PEI interaction. Consequently, higher CNF concentrations can still reduce the gelation time in alkaline environments by leveraging physical cross-links and enhancing the gel structure.

Higher CNF concentrations may promote faster gelation due to increased physical cross-linking at low and high pH, while at neutral pH, increased electrostatic repulsion may negate the effect of higher CNF concentrations, potentially slowing gelation.

**Table 7:** Sydansk code at extreme acidity (pH 2–3).

Time	1 wt% CNF	2 wt% CNF	3 wt% CNF	4 wt% CNF
24 h	C	D	E	E
48 h	D	F	F	G
72 h	D	F	G	H

**Table 8:** Sydansk code at extreme alkalinity (pH 13–14).

Time	1 wt% CNF	2 wt% CNF	3 wt% CNF	4 wt% CNF
24 h	A	B	B	B
48 h	A	B	B	B
72 h	B	B	B	B

**Table 9:** Sydansk code at neutral condition.

Time	1 wt% CNF	2 wt% CNF	3 wt% CNF	4 wt% CNF
24 h	I	I	E	H
48 h	I	I	H	H
72 h	I	I	I	I

### 2.2. Gel Strength

The sufficient strength of a polymer gel is another important component that impacts the effectiveness of oilfield water control application, in addition to gelation time. A polymer gel’s strength is a key factor in determining the range of deformation it can withstand without changing structurally. A thorough understanding of polymer gel’s strength can be attained via rheological analysis [15]. The benefit of this approach is that the gel structure can be preserved during the test, resulting in a precise and trustworthy result.

#### 2.2.1. Frequency Sweep

The frequency sweep studies were performed to obtain insight into how temperature, salinity, and pH impact the PAM/PEI polymer gel’s behavior rheologically at different CNF concentrations. Comprehending the impact of colloidal structure and dynamics, together with the stability and interplay between the three-dimensional networks inside the polymer gel, is crucial.

##### Effect of Temperature

**Figure 1:**
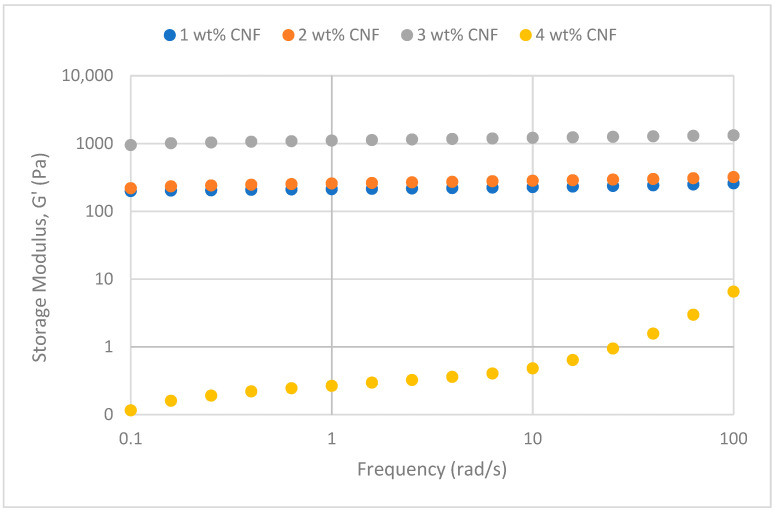
Storage modulus of frequency sweep at 70 °C.

**Figure 2:**
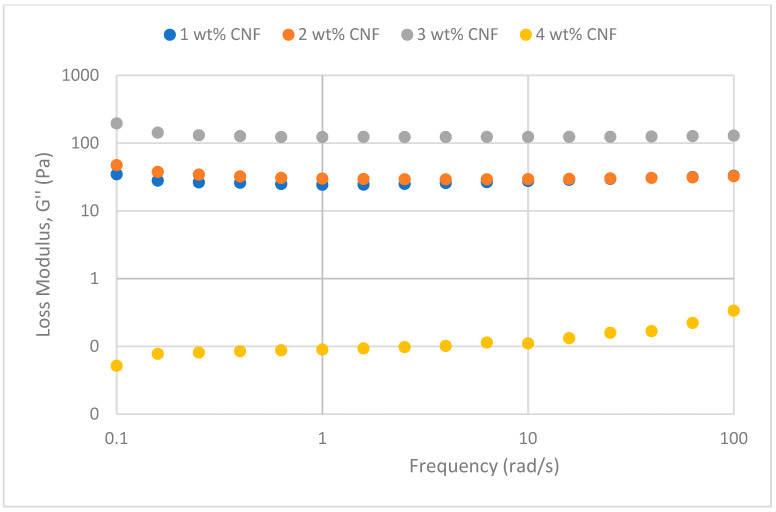
Loss modulus of frequency sweep at 70 °C.

**Figure 3:**
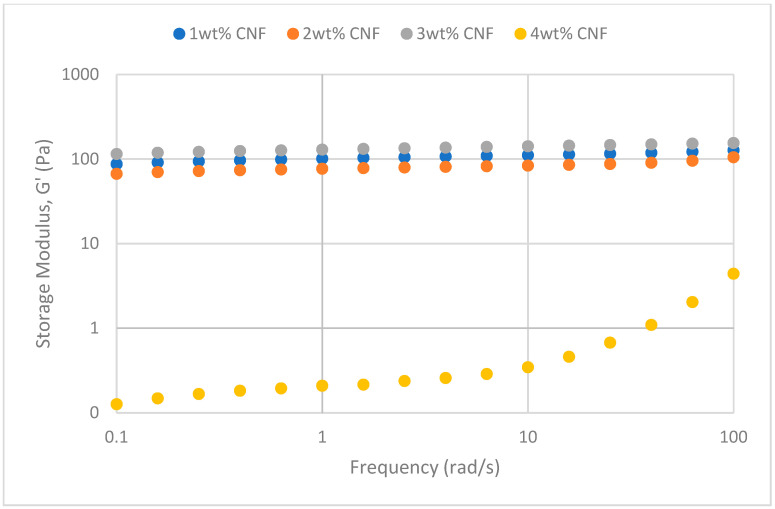
Storage modulus of frequency sweep at 80 °C.

**Figure 4:**
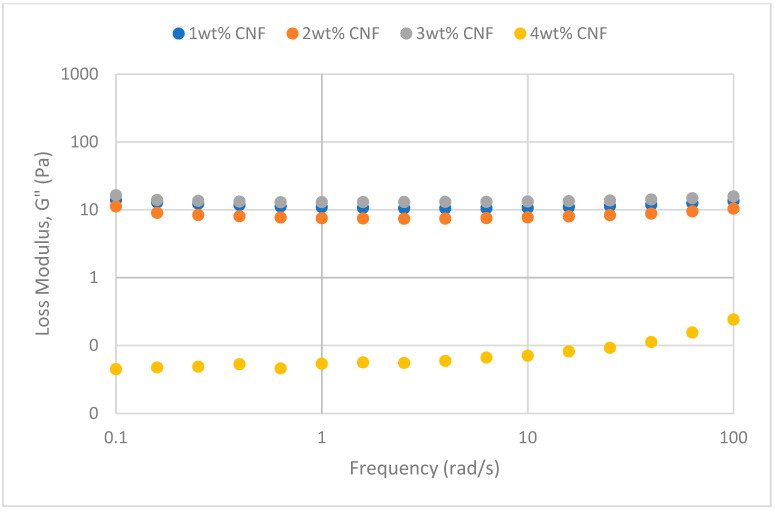
Loss modulus of frequency sweep at 80 °C.

**Figure 5:**
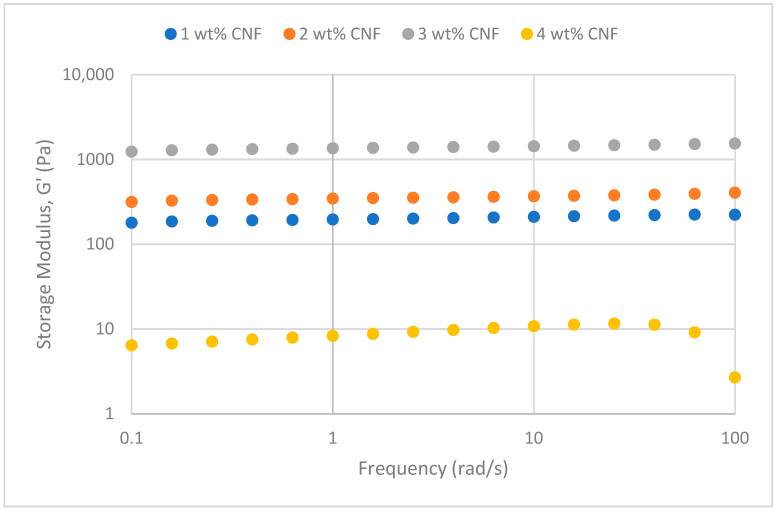
Storage modulus of frequency sweep at 90 °C.

**Figure 6:**
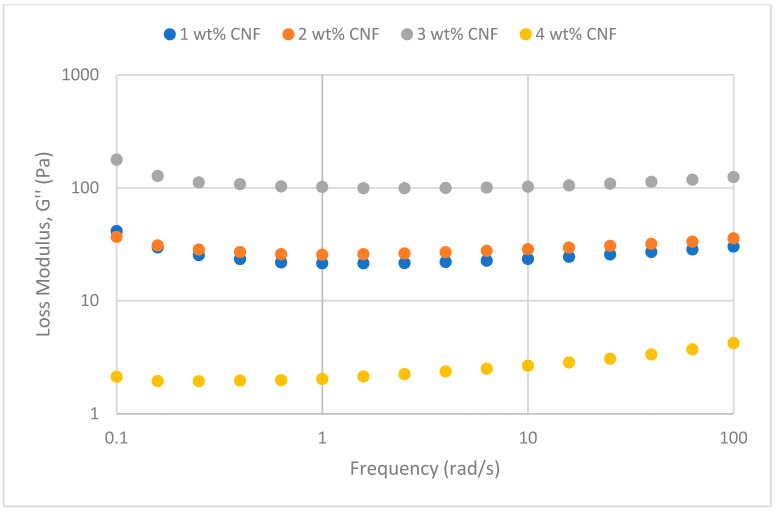
Loss modulus of frequency sweep at 90 °C.

The frequency sweeps dynamic rheological data for G′ and G″ at 70 °C, 80 °C, and 90 °C are presented in Figure 1, Figure 2, Figure 3, Figure 4, Figure 5 and Figure 6, respectively. From the figures, it is evident that all the values of storage modulus, G′, are larger than the loss modulus, G″, throughout the entire studied frequency range. This outcome demonstrates the elastic gel character predominately. Cellulose nanofibrils (CNF) and cross-linked polymer networks, such as PAM and PEI, aid in the formation of a robust and well-connected gel structure in CNF-PAM-PEI gels. With a mainly elastic behavior made possible by this structure, the gel has a larger storage modulus than the loss modulus.

As the CNF concentration increases, the gel strength (G′ and G″) also increases, with increasing frequency. The fibrous structure of CNF has a large aspect ratio and innate strength [16]. CNF serve as a reinforcement agent when integrated into the gel, improving the structural integrity of the gel network. The creation of a stronger and more linked gel structure is facilitated by the tangling and interweaving of CNF within the gel matrix. The presence of CNF increases the number of cross-linking points and promotes better entanglement, resulting in a denser and more cohesive gel structure. The optimum CNF concentration is 3 wt% CNF. Beyond this concentration, the gel strength decreases. This non-monotonic behavior can be attributed to the overcrowding of CNF in the gel matrix, leading to congestion and excessive density. At higher concentrations, the excessive tangling of CNF disrupts the gel network formation, hindering proper entanglement and cross-linking, thereby reducing gel strength. Similar non-monotonic trends in rheological moduli have been observed in other studies, such as silica-reinforced gels and nanoclay composites, where excessive filler concentrations resulted in gel destabilization due to phase separation or interference with the polymer network.

As the temperature increases, the gel strength (G′ and G″) consequently increases, with increasing frequency. The gel formulation at 90 °C had the highest gel strength compared to the gels at lower temperatures. Stronger interactions between the polymer constituents, such as PAM and PEI, can be encouraged at higher temperatures. Increased molecule mobility and the ability to establish longer, stronger intermolecular connections like hydrogen bonds and electrostatic interactions are both made possible by elevated temperatures. The creation of a stronger gel network because of these improved polymer–polymer interactions leads to an increase in gel strength.

##### Effect of Salinity

**Figure 7:**
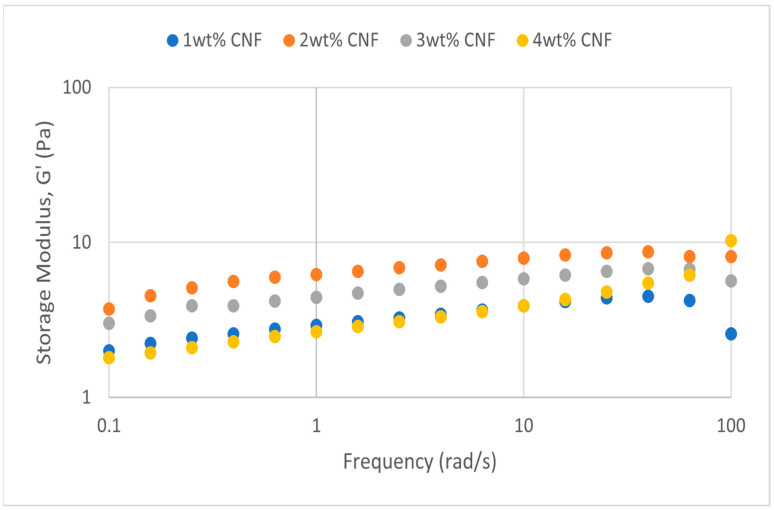
Storage modulus of frequency sweep at 30,000 ppm NaCl.

**Figure 8:**
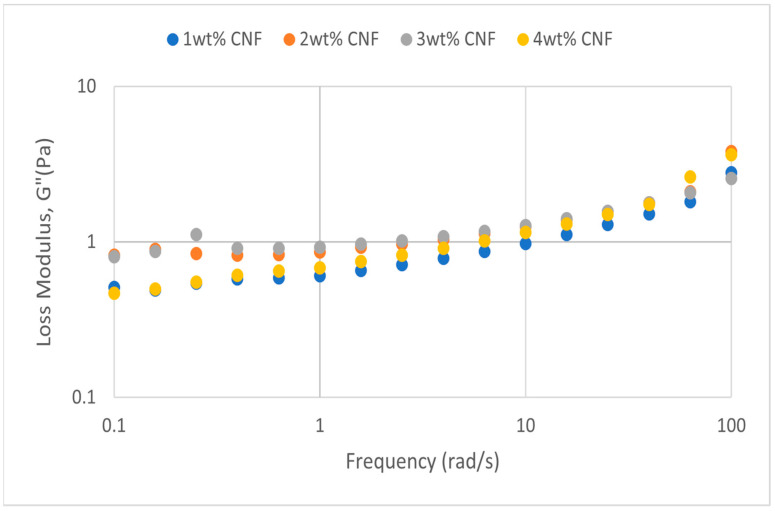
Loss modulus of frequency sweep at 30,000 ppm NaCl.

**Figure 9:**
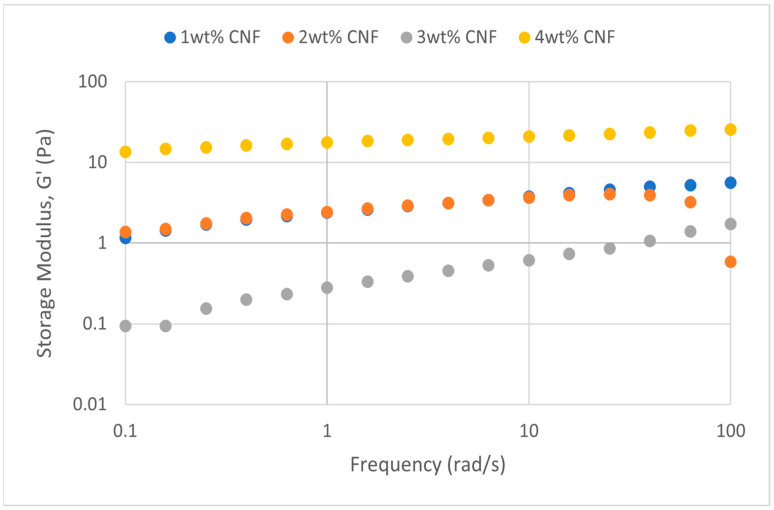
Storage modulus of frequency sweep at 40,000 ppm NaCl.

**Figure 10:**
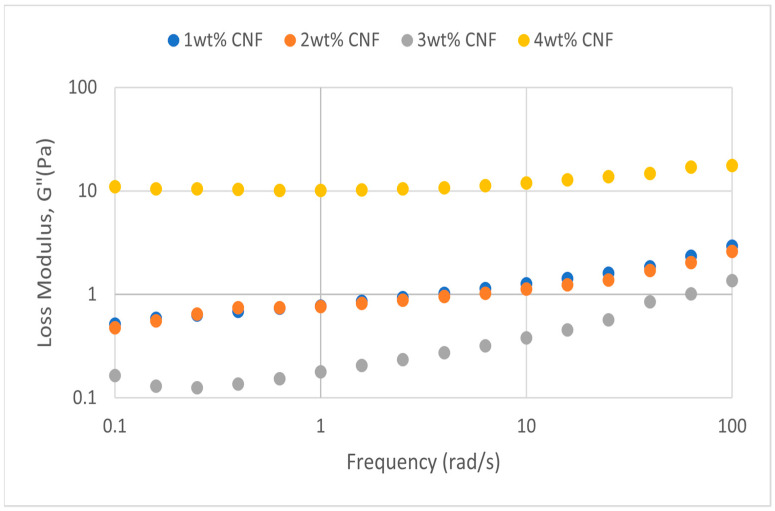
Loss modulus of frequency sweep at 40,000 ppm NaCl.

**Figure 11:**
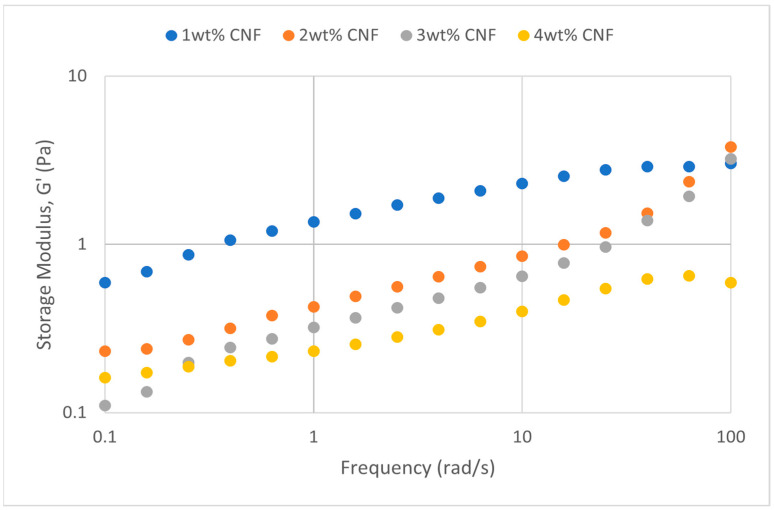
Storage modulus of frequency sweep at 50,000 ppm NaCl.

**Figure 12:**
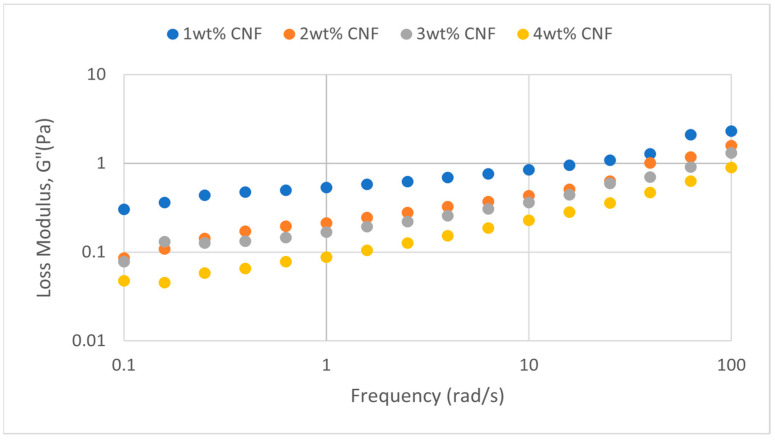
Loss modulus of frequency sweep at 50,000 ppm NaCl.

At various salinity, the frequency sweeps dynamic rheological data G′ and G″ are plotted against the oscillation frequency in Figure 7, Figure 8, Figure 9, Figure 10, Figure 11 and Figure 12, respectively.

It is clear from the figures that for the whole frequency range under study, the storage modulus (G′) is greater than the loss modulus (G″) in all tested conditions, indicating a predominantly elastic gel character. The strong and well-connected gel structure in a polymer gel, facilitated by cellulose nanofibrils (CNF) and cross-linked polymer networks, contributes to this elastic behavior. This trend is consistent across various salinities and pH conditions, demonstrating the existence of a fully formed network in every polymer gel, irrespective of the differing conditions. The higher storage modulus compared to the loss modulus underscores the elastic dominance in the gel’s mechanical properties.

Polymer gels exhibit elastic dominance over the examined frequency range at low salinities. However, as salinity rises, both G′ and G″ decrease, indicating a significant loss of viscoelastic behavior. The shift of the loss modulus, G″, toward higher frequencies with increasing salinity further underscores the detrimental impact on elasticity. This effect is due to the compression of electrical repulsion among negatively charged groups on the polymer backbone, causing the polymer chains to coil and compact. This compression prevents the polymer molecules from fully stretching, reducing the cross-linking process. Consequently, G″ values drop as the polymer chain aggregations are disrupted, and G′ abruptly decreases. The diffused electric double layer of polymer molecules is compressed by the salt ions, leading to a shrunken gel structure. High salinity results in compact aggregates due to strong associations, reducing aggregate sizes and weakening the elastic character. This reduction in viscoelasticity is due to fewer intermolecular bonds and less network structure formation, contributing to decreased elasticity in PAM/PEI polymer gel.

The frequency of the increase in gel strength (G′ and G″) is proportional to the concentration of CNF. The fibrous structure of CNF has an important aspect ratio and intrinsic strength [16]. When incorporated into the gel, CNF act as a reinforcing agent, enhancing the gel network’s structural stability. Entanglement and interweaving of CNF inside the gel matrix facilitates the formation of a stronger and more connected gel structure. A denser and more cohesive gel structure is produced when CNF are present because it enhances entanglement and increases the number of cross-linking points.

A 3 wt% of CNF is the ideal CNF concentration. Gel congestion causes the gel strength to drop below this concentration. Higher quantities of CNF may cause the gel network to become unusually dense and clogged. This overcrowding can impede the development of a networked gel structure as well as the tangling of CNF. The gel strength may plateau or even decrease because of the high concentration of CNF interfering with the gelation process.

The rheological behavior of PAM-PEI polymer gels at varying salinity levels shows distinct trends depending on the CNF concentration. The discrepancies observed in these trends may be attributed to the complex interplay between salinity, polymer chain behavior, and CNF dispersion. At higher salinity levels, the increased ionic strength compresses the electric double layer around the polymer chains, leading to coiling and reduced cross-linking efficiency. This effect can vary with CNF concentration, as CNF act as a physical cross-linker but may also agglomerate at higher concentrations, disrupting the gel network. Additionally, the increase in viscosity caused by higher CNF concentration might limit polymer mobility and the formation of uniform cross-links under high-salinity conditions. These mechanisms provide a plausible explanation for the observed non-linear trends in Figure 7, Figure 8, Figure 9, Figure 10, Figure 11 and Figure 12.

##### Effect of pH

**Figure 13:**
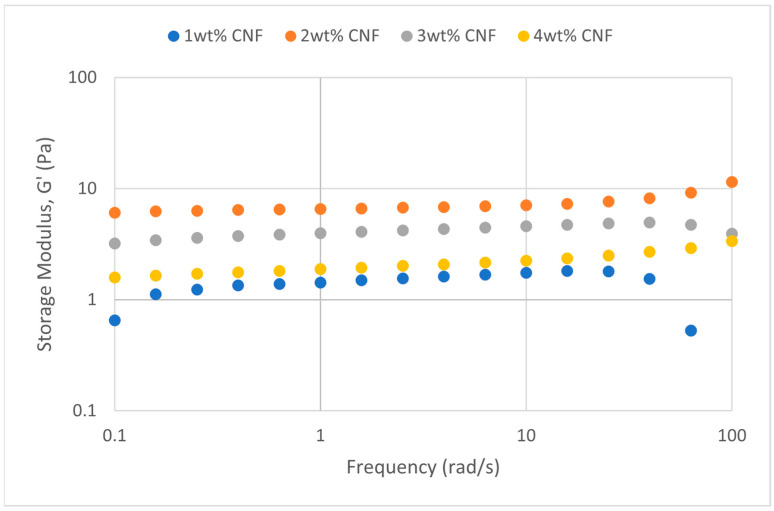
Storage modulus of frequency sweep at extreme acidity (pH 2–3).

**Figure 14:**
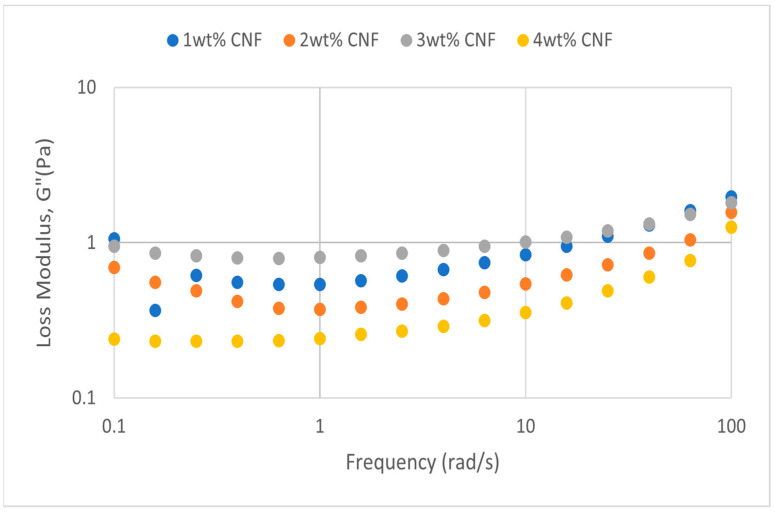
Loss modulus of frequency sweep at extreme acidity (pH 2–3).

**Figure 15:**
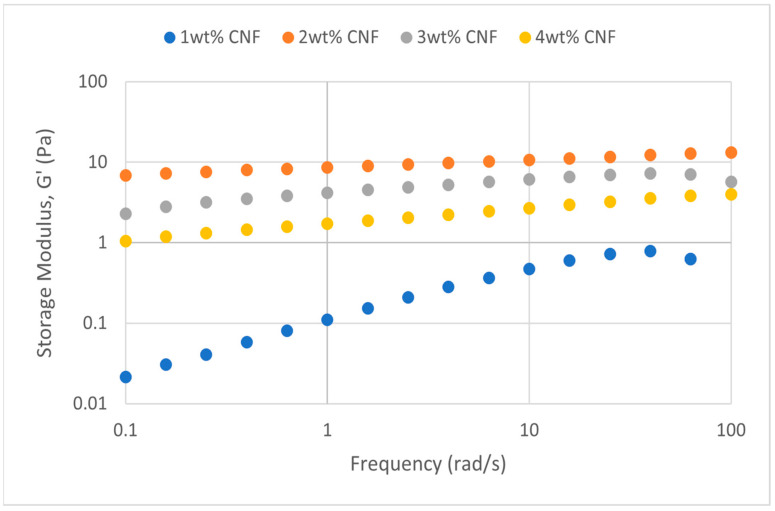
Storage modulus of frequency sweep at extreme alkalinity (pH 13–14).

**Figure 16:**
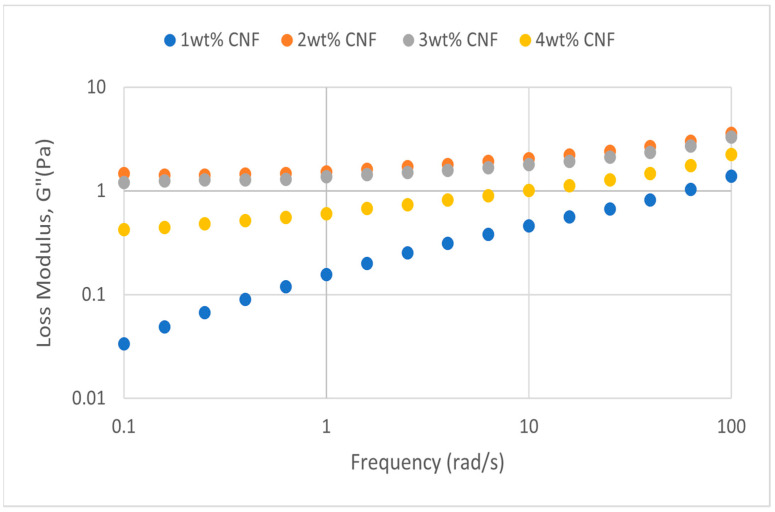
Loss modulus of frequency sweep at extreme alkalinity (pH 13–14).

**Figure 17:**
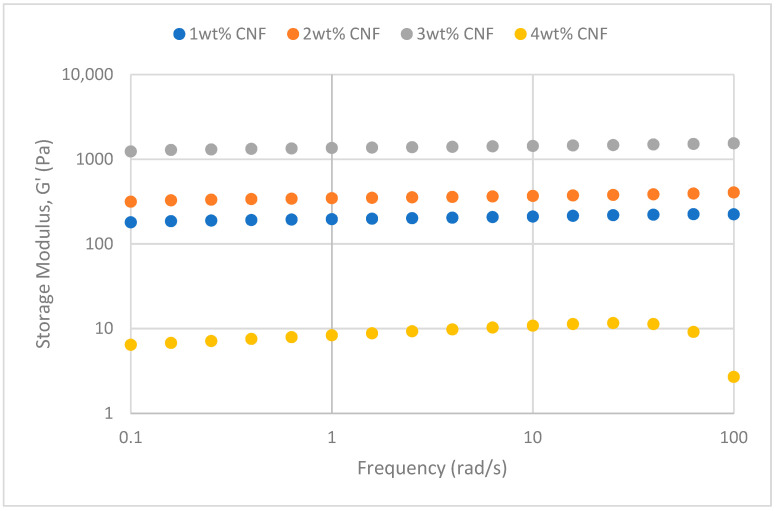
Storage modulus of frequency sweep at neutral condition.

**Figure 18:**
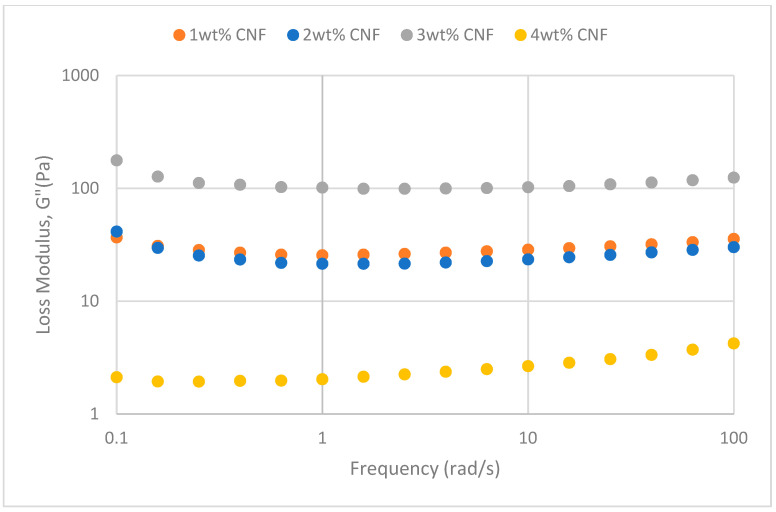
Loss modulus of frequency sweep at neutral condition.

The frequency sweep data for acidic, alkaline, and neutral conditions at different pH values provide crucial information about the behavior and gel strength of the PAM-PEI cross-linked polymer gel that was added with CNF. G′ and G″ are shown to drop when pH approaches the extremes, where it is either strongly alkaline (pH 13–14) or severely acidic (pH 2–3). This pattern suggests that at very high pH values, the gel’s viscoelastic qualities are weakened. At the acidic, neutral, and alkaline conditions, the frequency sweeps dynamic rheological data G′ and G″ are plotted against the oscillation frequency in Figure 13, Figure 14, Figure 15, Figure 16, Figure 17 and Figure 18, respectively.

The polymer gel has the highest values for G′ and G″ at neutral pH, indicating that the gel network is the most stable and long-lasting in these conditions. This stability may be facilitated by PEI’s protonation, which preserves equilibrium in its interactions with PAM and CNF and encourages the best possible cross-linking and network creation. On the other hand, the protonation of PEI increases its positive charge density at pH 2–3, increasing its interactions with less negatively charged CNF and PAM. However, as the severe acidity weakens the gel structure and lowers both G′ and G″ values, the overall strength of the network decreases. Similarly, weaker connections and greater repulsion among the polymer chains occur at pH 13, where the minimal protonation of PEI and high negative charge on CNF further reduce the gel strength.

Even at these extreme pH levels, an increase in CNF concentration leads to higher values of both moduli despite the general drop in G′ and G″ under these conditions. This suggests that by strengthening the gel network, greater CNF concentrations can somewhat counteract the adverse effects of extremely high pH. The extra physical cross-links provided by CNF improve the gel’s flexibility and structural integrity. Consequently, although the gel is naturally weakened by high pH values, this impact is counteracted by raising the CNF content, which strengthens the gel’s overall network structure. The increase in gel strength decreases after 3wt% CNF, suggesting a saturation limit at which more CNF do not have much effect on subsequent cross-linking or network stability. There are multiple reasons for the gel strength plateau that was seen above 3 wt% CNF. First off, there might be a limit to the physical cross-linking capacity of CNF, after which more CNF do not considerably enhance the creation of new cross-links. Second, CNF may begin to agglomerate at larger concentrations, which may impair its capacity to evenly reinforce the gel network. Lastly, too much CNF may make the solution more viscous, which would hinder polymer chain mobility and interaction and prevent future gel strength gains.

The effects of pH on the rheological properties of PAM-PEI polymer gels highlight significant variations across acidic, neutral, and alkaline conditions. At acidic pH, the protonation of PEI increases its positive charge density, enhancing interactions with PAM and CNF. However, higher CNF concentrations lead to increased viscosity, potentially limiting polymer mobility and hindering the formation of a cohesive network. In contrast, at alkaline pH, deprotonation of PEI reduces its charge density, weakening interactions with PAM. Fully dissociated carboxylate groups on CNF may also introduce repulsive forces, further disrupting the gel network. These factors explain the non-linear trends in gel strength and modulus observed at different pH levels. At neutral pH, the balance of charges between PEI, PAM, and CNF results in the most stable gel network, reflected in the highest G′ and G″ values.

#### 2.2.2. Strain Sweep

##### Effect of Temperature

**Figure 19:**
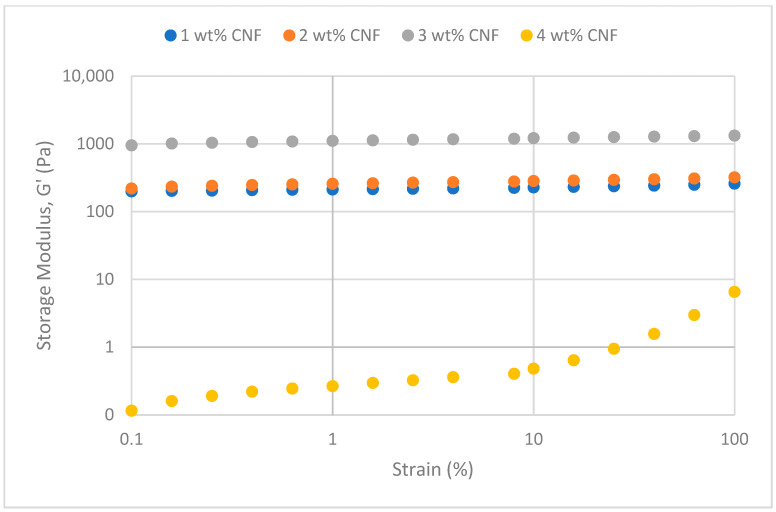
Storage modulus of strain sweep at 70 °C.

**Figure 20:**
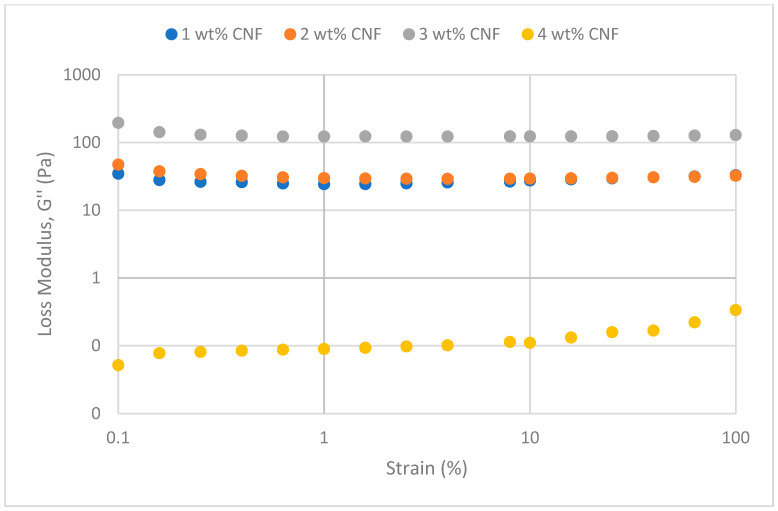
Loss modulus of strain sweep at 70 °C.

**Figure 21:**
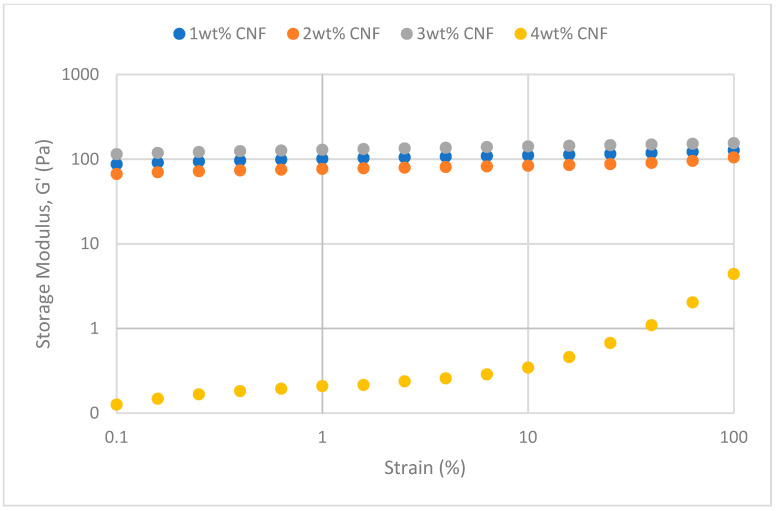
Storage modulus of strain sweep at 80 °C.

**Figure 22:**
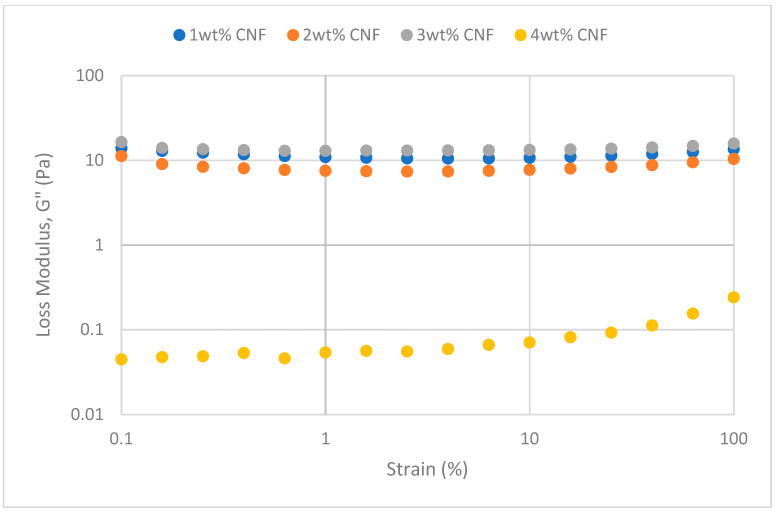
Loss modulus of strain sweep at 80 °C.

**Figure 23:**
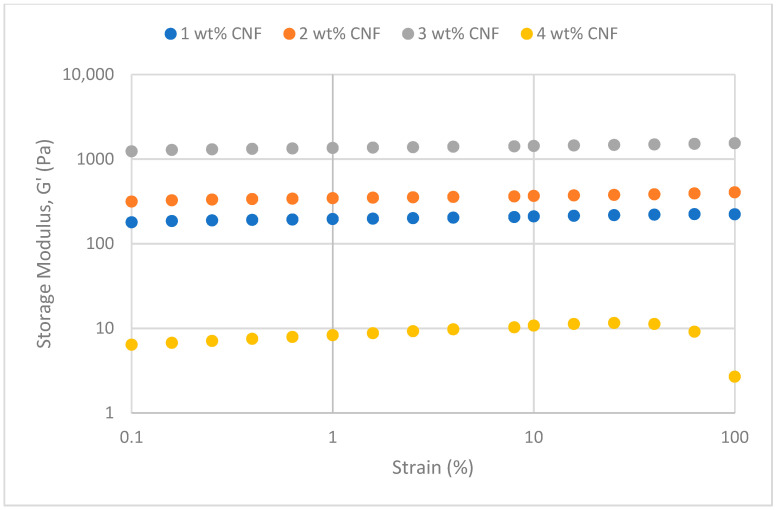
Storage modulus of strain sweep at 90 °C.

**Figure 24:**
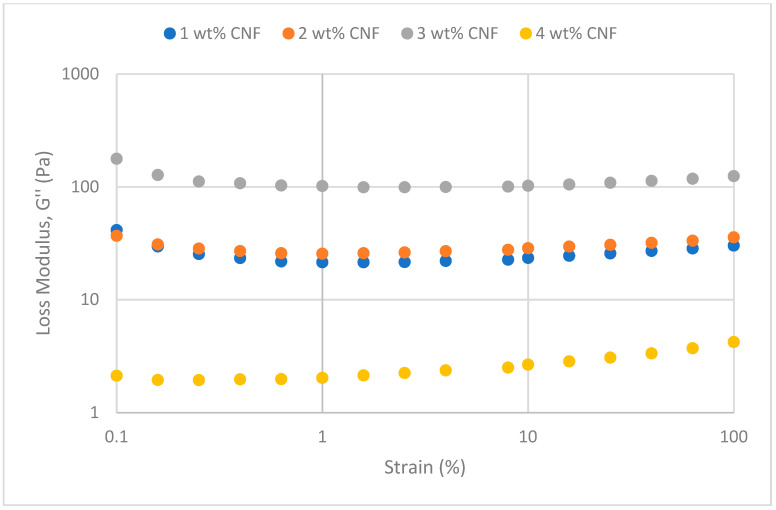
Loss modulus of strain sweep at 90 °C.

The strain sweeps dynamic rheological data for G′ and G″ at 70 °C, 80 °C, and 90 °C are presented in Figure 19, Figure 20, Figure 21, Figure 22, Figure 23 and Figure 24, respectively. From the figures, it is evident that all the values of storage modulus, G′, are larger than the loss modulus, G″, throughout the entire studied strain range. This outcome demonstrates the elastic gel character predominately. Cellulose nanofibrils (CNF) and cross-linked polymer networks, such as PAM and PEI, aid in the formation of a robust and well-connected gel structure in CNF-PAM-PEI gels. With a mainly elastic behavior made possible by this structure, the gel has a larger storage modulus than the loss modulus.

As the CNF concentration increases, the gel strength (G′ and G″) also increases, with increasing strain. Hydrogen bonds and other interactions, such as PAM and PEI, can be formed between CNF and the polymer matrix [16]. These interactions add to the gel network’s connection points, strengthening intermolecular forces and enhancing gel strength. The presence of CNF increases the number of cross-linking points and promotes better entanglement, resulting in a denser and more cohesive gel structure. The optimum CNF concentration is 3 wt% CNF. Beyond this concentration, the gel strength decreases due to gel overcrowding. The gel network may become congested and abnormally dense at greater CNF concentrations. The tangling of CNF and the development of a networked gel structure can both be hampered by this overpopulation. Due to the high CNF concentration interference with the gelation process, the gel strength tends to drop.

As the temperature increases, the gel strength (G′ and G″) consequently increases, with increasing strain. The gel formulation at 90 °C had the highest gel strength compared to the gels at lower temperatures. The gel matrix’s polymer chains become more mobile and flexible as the temperature rises. Due to the polymer chains’ improved ability to entangle and interlock with one another due to their greater mobility, the gel structure becomes thicker and more linked. The strength and resistance to deformation of the gel are enhanced by the entanglement of polymer chains.

##### Effect of Salinity

**Figure 25:**
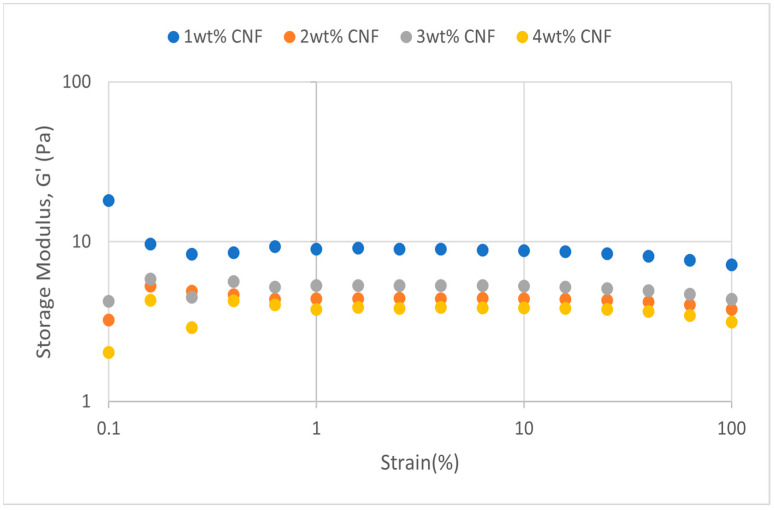
Storage modulus of strain sweep at 30,000 ppm NaCl.

**Figure 26:**
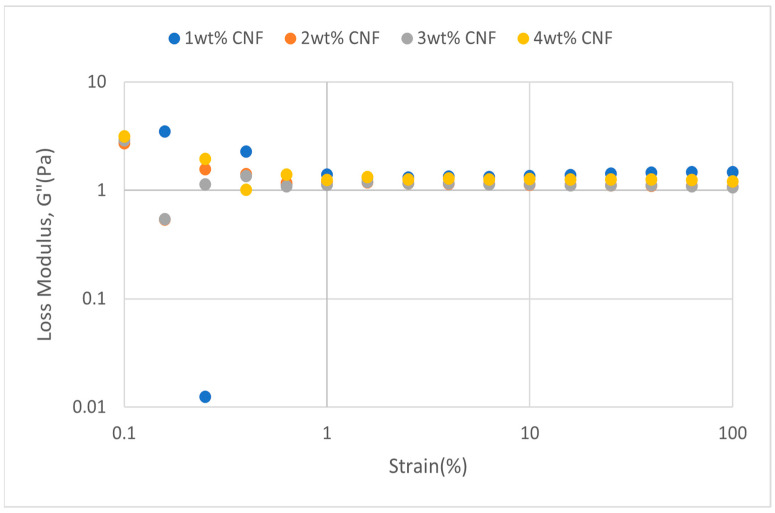
Loss modulus of strain sweep at 30,000 ppm NaCl.

**Figure 27:**
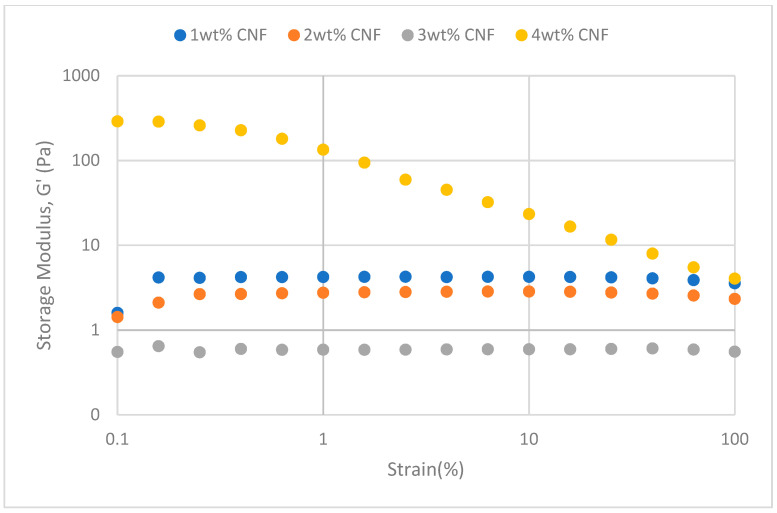
Storage modulus of strain sweep at 40,000 ppm NaCl.

**Figure 28:**
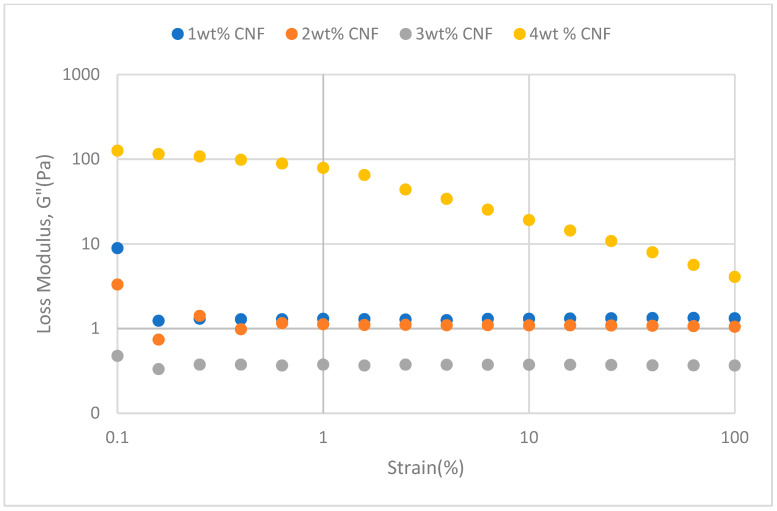
Loss modulus of strain sweep at 40,000 ppm NaCl.

**Figure 29:**
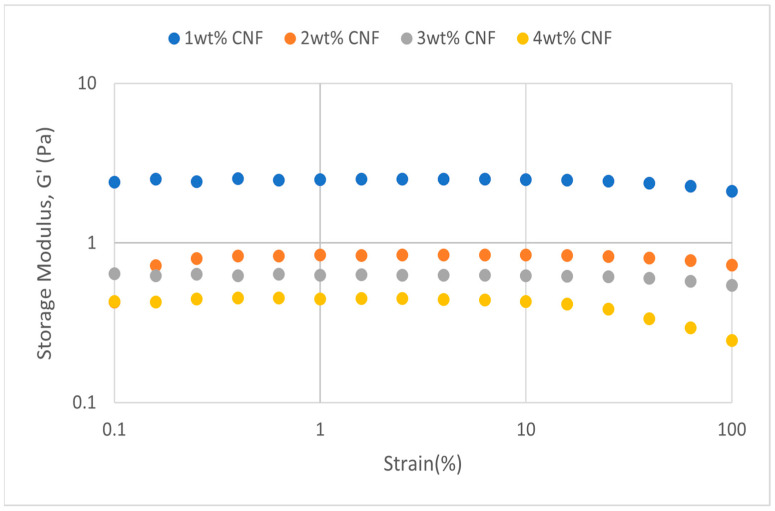
Storage modulus of strain sweep at 50,000 ppm NaCl.

**Figure 30:**
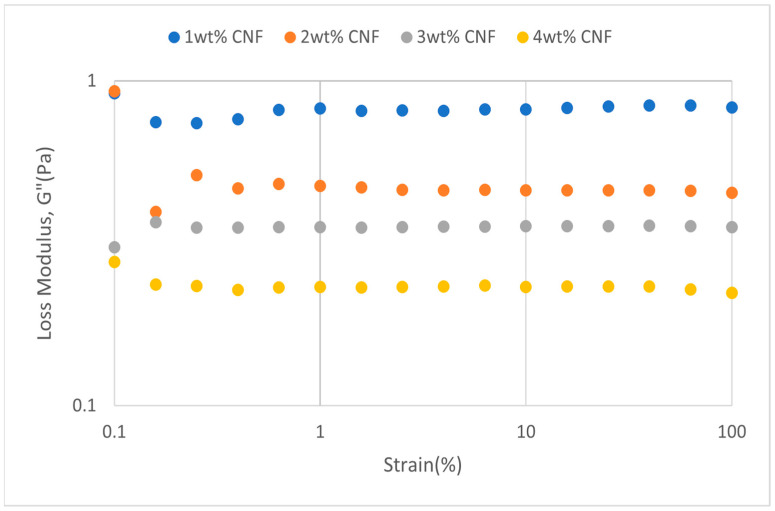
Loss modulus of strain sweep at 50,000 ppm NaCl.

The oscillation’s strains are varied at a fixed frequency for strain sweeps to quantify the moduli. Viscosity behavior will predominate in solution behavior when the sample’s structural network is torn apart by strain exceeding the crucial threshold value. The solution is more flexible when the yield point is bigger [17]. At various salinity, the strain sweeps dynamic rheological data G′ and G″ are plotted against the oscillation frequency in Figure 25, Figure 26, Figure 27, Figure 28, Figure 29 and Figure 30, respectively.

From the figures, it is clear that under all tested conditions and over the whole analyzed frequency range, the storage modulus (G′) is greater than the loss modulus (G″), indicating a predominantly elastic gel character. The strong and well-connected gel structure in a polymer gel, facilitated by cellulose nanofibrils (CNF) and cross-linked polymer networks, contributes to this elastic behavior. This trend is consistent across various salinities and pH conditions, demonstrating the existence of a fully formed network in every polymer gel, irrespective of the differing conditions. The higher storage modulus compared to the loss modulus underscores the elastic dominance in the gel’s mechanical properties.

As the concentration of NaCl increases, both G′ and G″ decrease, indicating a significant loss of viscoelastic behavior. The compression of the electrical double layer around the polymer chains by salt ions causes the polymer chains to coil and compact. This compression prevents the polymer molecules from fully stretching and reduces the cross-linking process. Consequently, G″ values drop as the polymer chain aggregations are disrupted, and G′ abruptly decreases. High salinity results in compact aggregates due to strong associations, reducing aggregate sizes and weakening the elastic character. This reduction in viscoelasticity is due to fewer intermolecular bonds and less network structure formation, contributing to decreased elasticity in PAM/PEI polymer gel.

The gel strengths (G′ and G″) rise with increasing CNF concentration up to 2 wt% of CNF under increasing strain. The fibrous structure of CNF has an important aspect ratio and intrinsic strength [16]. When incorporated into the gel, CNF act as a reinforcing agent, enhancing the gel network’s structural stability. Entanglement and interweaving of CNF inside the gel matrix facilitates the formation of a stronger and more connected gel structure. A denser and more cohesive gel structure is produced when CNF are present because it enhances entanglement and increases the number of cross-linking points.

The trends in G′ and G″ vary depending on the experimental conditions, as indicated in the data. Figure 25 suggests that the modulus at 1 wt% CNF is the highest, while Figure 27 shows the highest values at 4 wt% CNF. This indicates that the optimal CNF concentration for maximizing gel strength may depend on specific factors, such as the interaction between the polymer matrix and CNF under varying conditions. For a well-balanced gel network, a CNF concentration of 2 wt% would reach the maximum for both G′ and G″. The cellulose nanofibrils (CNF) contribute to additional physical cross-links within the polymer matrix, enhancing the cohesiveness and interconnectedness of the gel structure. However, at higher CNF concentrations, potential overcrowding and agglomeration might impede the mobility and interaction of polymer chains, leading to a plateau or slight reduction in gel strength at 50,000 ppm salinity.

##### Effect of pH

**Figure 31:**
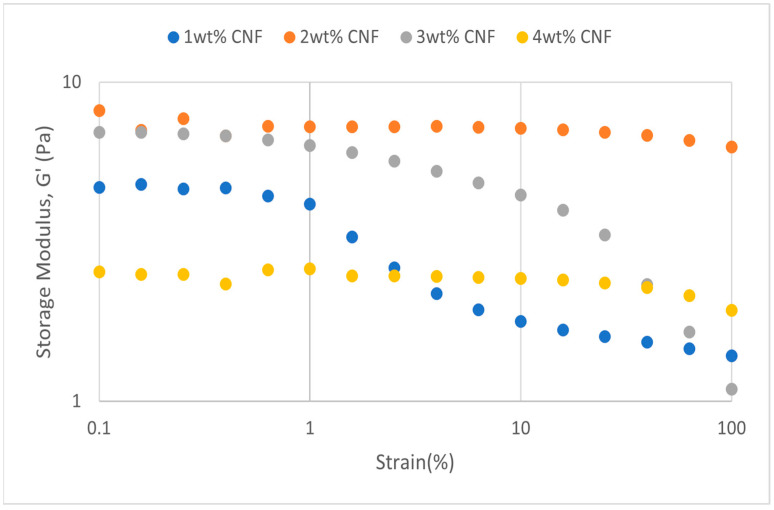
Storage modulus of strain sweep at extreme acidity (pH 2–3).

**Figure 32:**
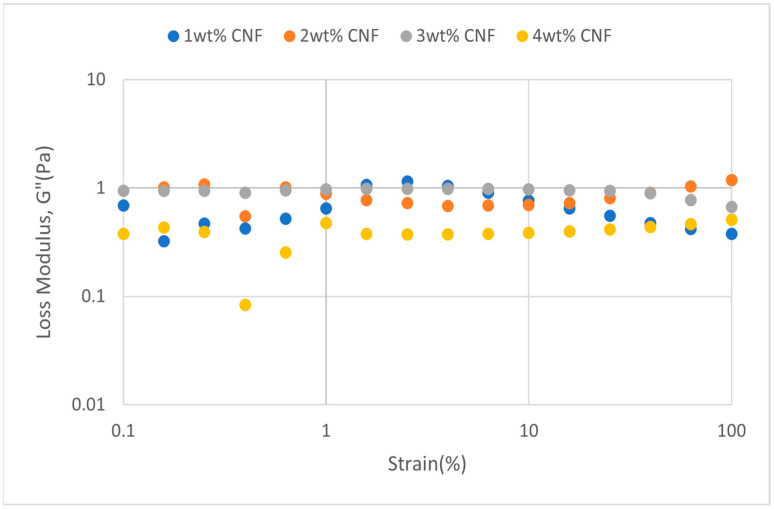
Loss modulus of strain sweep at extreme acidity (pH 2–3).

**Figure 33:**
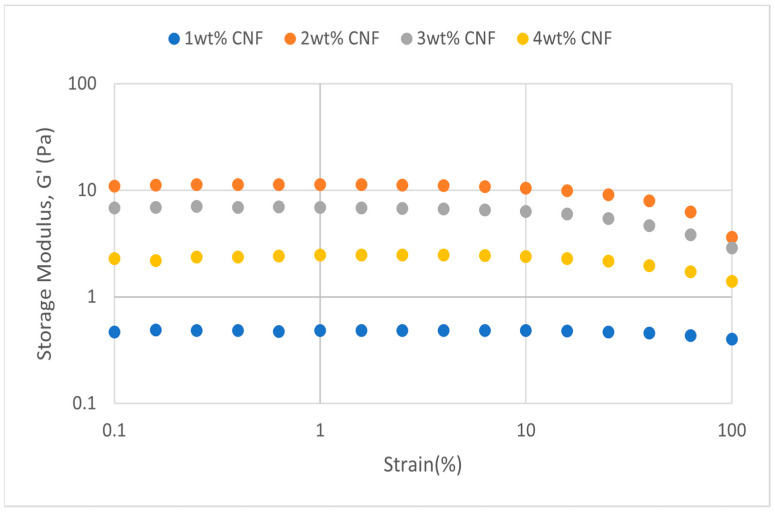
Storage modulus of strain sweep at extreme alkalinity (pH 13–14).

**Figure 34:**
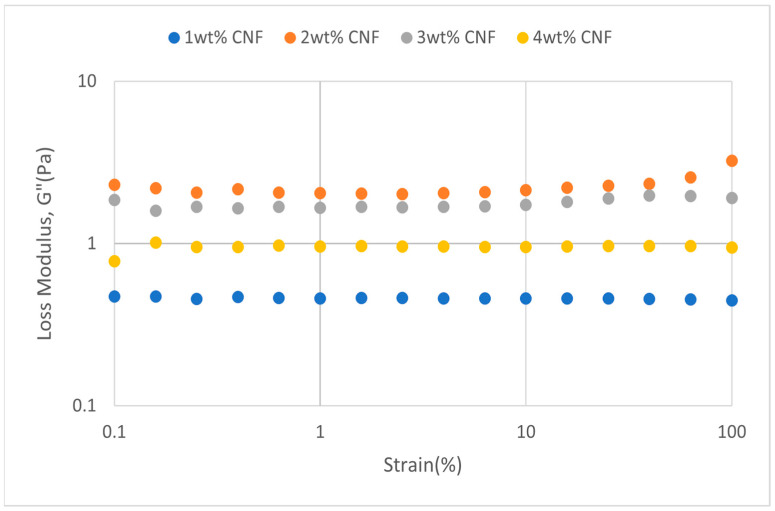
Loss modulus of strain sweep at extreme alkalinity (pH 13–14).

**Figure 35:**
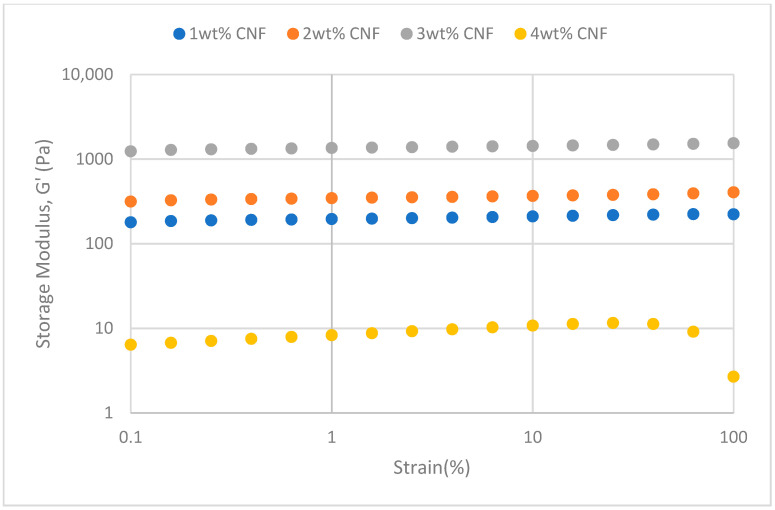
Storage modulus of strain sweep at neutral condition.

**Figure 36:**
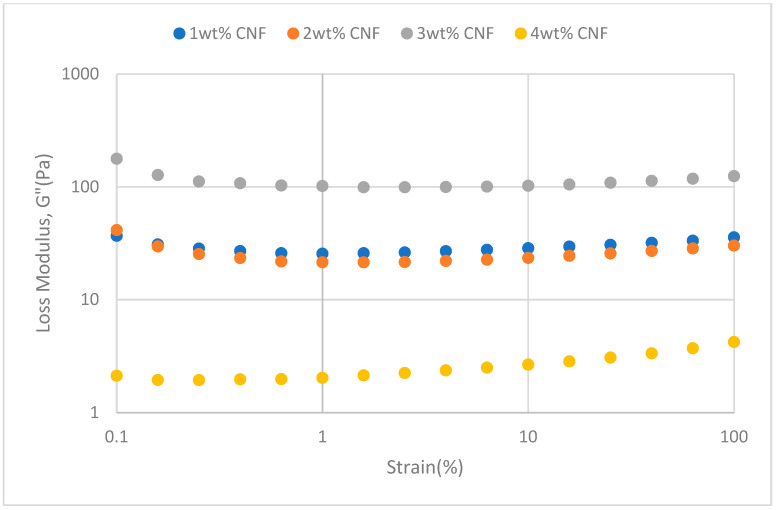
Loss modulus of strain sweep at neutral condition.

The strain sweep data for acidic, alkaline, and neutral conditions at different pH values provide crucial information about the behavior and gel strength of the PAM-PEI cross-linked polymer gel that was added with CNF. G′ and G″ are shown to drop when pH approaches the extremes, where it is either strongly alkaline (pH 13–14) or severely acidic (pH 2–3). This pattern suggests that at very high pH values, the gel’s viscoelastic qualities are weakened. At the acidic, neutral, and alkaline conditions, the strain sweeps dynamic rheological data G′ and G″ are plotted against the oscillation frequency in Figure 31, Figure 32, Figure 33, Figure 34, Figure 35 and Figure 36, respectively. The strain sweep data are consistent with the previous findings from the frequency sweep studies.

The polymer gel has the highest values for G′ and G″ at neutral pH, indicating that the gel network is the most stable and long-lasting in these conditions. This stability may be facilitated by PEI’s protonation, which preserves equilibrium in its interactions with PAM and CNF and encourages the best possible cross-linking and network creation. On the other hand, the protonation of PEI increases its positive charge density at pH 2–3, increasing its interactions with less negatively charged CNF and PAM. However, as the severe acidity weakens the gel structure and lowers both G′ and G″ values, the overall strength of the network decreases. Similarly, weaker connections and greater repulsion among the polymer chains occur at pH 13, where the minimal protonation of PEI and high negative charge on CNF further reduce the gel strength.

Even at these extreme pH levels, an increase in CNF concentration leads to higher values of both moduli despite the general drop in G′ and G″ under these conditions. This suggests that by strengthening the gel network, greater CNF concentrations can somewhat counteract the adverse effects of extremely high pH. The extra physical cross-links provided by CNF improve the gel’s flexibility and structural integrity. Consequently, although the gel is naturally weakened by high pH values, this impact is counteracted by raising the CNF content, which strengthens the gel’s overall network structure.

The increase in gel strength decreases after 2 wt% CNF, suggesting a saturation limit at which more CNF do not have much effect on subsequent cross-linking or network stability. There are multiple reasons for the gel strength plateau that was seen above 2 wt% CNF. First off, there might be a limit to the physical cross-linking capacity of CNF, after which more CNF do not considerably enhance the creation of new cross-links. Second, CNF may begin to agglomerate at larger concentrations, which may impair its capacity to evenly reinforce the gel network. Lastly, too much CNF may make the solution more viscous, which would hinder polymer chain mobility and interaction and prevent future gel strength gains.

## 3. Conclusions

The effect of incorporating nanocellulose into polymer gel on gel strength and gelation time at different temperatures, salinity, and pH levels has been examined in this study. The addition of CNF to synthetic polymer gel considerably improves its mechanical strength and stability, indicating that it may be a useful addition for controlling oilfield water. The results of the experiment demonstrate that lower temperature, increased salinity, and extreme pHs lengthen the gelation period and reduce the gel’s strength. High salinity (50,000 ppm) reduced gel strength by compressing polymer chains, with higher CNF concentration potentially contributing to agglomeration and increased viscosity, which limited polymer chain mobility and cross-linking efficiency. At extreme pH conditions, the behavior of CNF and their interaction with PAM and PEI depended on protonation and charge balance, with acidic and alkaline conditions disrupting gel network formation due to increased repulsive forces or viscosity effects at higher CNF concentration. Neutral pH provided the optimal conditions for gel stability and strength due to balanced interactions among the components. The key findings are that increasing the concentration of CNF further elongates the gelation time at 90 °C, 50,000 ppm NaCl salinity, and shortens it at a pH of 2–3 and pH 13–14. CNF also strengthened the gel at high temperatures, salinity, and extreme pH. The CNF concentration of 2 wt% to 3 wt% are optimum to yield the best result for longer gelation time and stronger polymer gel. Hence, CNF improved the PAM-PEI cross-linked polymer gel, which was able to withstand diverse reservoir conditions in the mitigation of excessive water production. To further develop and optimize the nanocellulose-improved polymer gel, future work should focus on understanding the agglomeration behavior of CNF under varying conditions and its role in influencing the viscoelastic properties of polymer gels. Additionally, exploring alternative cross-linking strategies or modified CNF structures could enhance gel performance under extreme reservoir conditions.

## 4. Materials and Methods

### 4.1. Materials

Polyethylenimine (PEI), a liquid polymer with a molecular weight of 35,000 g/mol and an active content of 99%, was used as the cross-linker in this study. It was supplied by BASF and used as received without further processing. Non-ionic polyacrylamide (PAM), a granular, straight-chain polymer made of acrylamide monomers, was utilized as the polymer. PAM, with a molecular weight of 5–6 million g/mol, was purchased from Sigma Aldrich and used without further modification. Cellulose nanofibrils (CNF), a nanocellulose material with a high aspect ratio, were employed as the reinforcement material in the PAM-PEI gel formulation. CNF, typically having a diameter of 5–20 nm and a length ranging from 100 nm to several micrometers, were obtained from a specialized supplier. CNF have a high surface area (around 150–250 m^2^/g) and excellent mechanical properties, making them an ideal reinforcement material. Sodium chloride (NaCl), used to adjust the salinity conditions for the tests, was purchased from Sigma Aldrich (M) Sdn Bhd, Selangor, Malaysia (ACS grade). NaCl has a molecular weight of 58.44 g/mol and a melting point of 801 °C. Deionized water, produced in our laboratory, was used as the solvent for CNF and in the preparation of all solutions and gels. The resistivity of the DI water was measured to be approximately 18.2 MΩ·cm, which is typical for high-purity deionized water.

### 4.2. Preparation of Polymer Gel

First, a combination was made by mixing 1 mL of PEI with 0.25 g of PAM [18]. The PAM-PEI gel was then filled to a total volume of 50 mL with various concentrations of CNF (1 wt%, 2 wt%, 3 wt%, and 4 wt%) in deionized water. For the salinity test, 1.5 g, 2.0 g, and 2.5 g of NaCl were added to monitor its effects in the presence of CNF. To analyze the effect of pH, the mixture was adjusted to an acidic condition (pH 2–3) using HCl, a neutral condition, and an alkaline condition (pH 13–14) using NaOH. The mixture was stirred for one hour at room temperature using an IKA RW 20 Digital homogenizer by IKA Works (Asia) Sdn Bhd, Selangor, Malaysia at 300 rpm. The homogenizer used was a digital, mechanical type, which provides adequate shear forces for proper dispersion of the CNF without the heat generation issues associated with ultrasonic homogenizers. To ensure uniform dispersion of the CNF and prevent clumping, the mixture was continuously stirred. Afterward, the gel mixture was transferred into test tubes and placed into a water bath in the oven. The test tubes were kept in a high-temperature oven at various temperatures ranging from 70 °C to 90 °C for the temperature study, while the mixture with varying salinity and pH was maintained at 90 °C to form the gel [19].

### 4.3. Experimental Analysis

#### 4.3.1. Measuring Gelation Time

The bottle testing strategy is a semi-quantitative method used to determine the gel rate and strength. This method works well for assessing the long-term durability of gel samples at high temperatures and is also reasonably priced. The Sydansk gel code was used to compare gel rigidity, where the bottles were filled with equal volumes of the prepared gellant at the designated temperature [19]. The gels were monitored every 24 h for 3 days. The samples could be observed by periodically flipping the vial. Based on these findings, each sample was assigned a gel code [20]. The amount of time needed for the gel to become rigid is determined as the gelation time. The Sydansk code for the bottle test and its description are shown in Appendix A.

#### 4.3.2. Measuring Gel Strength

Important data regarding the strength and viscoelastic properties of polymer gels are gathered via the oscillation test. The oscillation or gel strength was assessed using an oscillation rheometer by TA Instruments, Selangor, Malaysia coupled with a parallel plate geometry measurement device. In this experiment, a small number of gel samples were placed on the rheometer between two parallel Peltier plate geometries, each with a 40 mm diameter and a fixed and rotating arrangement at a 4° angle. The chosen spacing between them was one millimeter. Room temperature was used for each measurement. The viscoelastic properties of the polymeric gel are indicated by the storage modulus (G′) and the loss modulus (G″). Typically, G″ reflects viscous behavior, whereas G′ predominantly signifies the elasticity of the gel system [15].

## Data Availability

The original contributions presented in this study are included in the article. Further inquiries can be directed to the corresponding author.

## Appendix A

**Gel Strength Code**	**Gel Description**
A	No detectable gel formed: The gel has the same viscosity as the initial polymer solution
B	Highly flowing gel: The gel doesn’t seem to be all that viscous, compared to the initial polymer solution
C	Flowing gel: Upon inversion, most of the gel flows by gravity to the bottle top
D	Moderately flowing gel: Upon inversion, only a small amount of the gel does not gravitationally flow to the bottle
E	Barely flowing gel: The gel can hardly flow to the bottle cap
F	Highly deformable nonflowing gel: When the bottle is inverted, the gel does not naturally flow to the bottle top
G	Moderately deformable nonflowing gel: Gravity causes the gel to deform approximately halfway down the bottle upon inversion
H	Slightly deformable nonflowing gel: When the gel is inverted, gravity just slightly distorts the gel surface
I	Rigid gel: Upon inversion, there is no gel surface deformation caused by gravity
J	Ringing rigid gel: When the bottle is tapped, a mechanical vibration resembling a tuning fork is audible
